# Ensemble of CMIP6 derived reference and potential evapotranspiration with radiative and advective components

**DOI:** 10.1038/s41597-023-02290-0

**Published:** 2023-06-27

**Authors:** Nels Bjarke, Joseph Barsugli, Ben Livneh

**Affiliations:** 1 Civil, Environmental, and Architectural Engineering Dept., University of Colorado, Boulder, USA; 2 Cooperative Institute for Research in Environmental Sciences, Boulder, USA; 3 NOAA Physical Science Laboratory, Boulder, USA

**Keywords:** Hydrology, Hydrology, Climate change

## Abstract

Assessing changes in future aridity requires an understanding of variations in the atmospheric demand for water. Such assessments are often driven by estimations of potential evapotranspiration (ET_P_) and/or reference evapotranspiration (ET_0_), yet no comprehensive and validated estimate of these climate metrics exists to date from the Coupled Model Intercomparison Project 6 (CMIP6). Here we describe the development and validation of a published dataset of global monthly estimates of the Penman-Monteith derived ET_0_, its advective and radiation components, Priestley-Taylor derived ET_P_, and vapor pressure deficit from 16 CMIP6 projections and four emissions scenarios. Historical validation of the ensemble of CMIP6 evaporative demand shows general agreement with observationally-derived baselines of ET_0_ and ET_P_ from the Climate Research Unit (CRU) and ERA5-Land reanalysis products, with GCM biases driven primarily by regional differences in modeled humidity and advective contributions to ET_0_. Overall, evaporative demand is projected to increase across all emissions scenarios, with the largest increases over polar regions, and with a larger contribution from advection particularly for regions with higher baseline ET_0_.

## Background & Summary

The evaporative demand of surface water to the atmosphere has been shown to be an essential driver and explanatory variable for numerous evaluations of changes in surface freshwater availability, agricultural water demand and drought risk^1–9^. Despite its ubiquitous use in analyses of changes in regional and global hydroclimates, there is a notable absence of an organized dataset of estimates of evaporative demand from the latest generation of Coupled Model Intercomparison Project 6 (CMIP6) General Circulation Models (GCMs). To address this need, we have developed monthly estimates of evaporative demand across a multi-model ensemble of CMIP6 GCM simulations These 16 GCMS are hereafter referred to as the CMIP6-MME.

Debate over the utility of the differing methods available for estimating evaporative demand^5,10–12^ and limitations in the availability of certain hydroclimate variables required for those estimations can lead to a large range of differing methodologies applied for the estimations of ET_P_^13^, ET_0_^14–16^, and VPD^16,17^. The purpose of this dataset^18^ is to serve as a tool for future hydroclimate analyses and to promote methodological consistency across studies that utilize future estimations of evaporative demand. To this end, we provide monthly estimations using two of the most commonly used methods: the Priestley-Taylor potential evapotranspiration (ET_P_)^13^ method, and the FAO-56 Penman-Monteith reference evapotranspiration (ET_0_)^16^ method for both short grass and alfalfa vegetation land-cover. We also provide a monthly estimate of a key driving variable, vapor pressure deficit (VPD) (Sec. 2.1), that is commonly used to assess ecological impacts. All quantities are provided for the historical simulation period (1850–2014) and four future (2015–2100) CMIP6 ScenarioMIP shared socioeconomic pathways (SSPs), 1–2.6, 2–4.5, 3–7.0, and 5–8.5^19,20^. These estimations are generated for the CMIP6-MME, of which ensemble members are selected based on the availability of all climate variables needed to complete the multiple estimations evaporative demand described below.

Following the development of the CMIP6-MME of ET_P_, ET_0_, and VPD, we perform a validation using observation-based historical baselines of both the monthly estimations of ET_0_ from the Climate Research Unit (CRU) dataset^21^ and estimations of ET_P_ and ET_0_ derived from the ERA5-Land reanalysis^22^. Along with the expected variation in performance due to differences in GCM simulated historical hydroclimates^23,24^, we note a general coherence in the bias that exists across all GCMs within both ET_P_ and ET_0_ that likely arise due the assumptions embedded within each methodology. Specifically, we evaluate differences the two major components evaporative demand, the advection and radiation components, from each GCM using the same components from ERA5-Land as the baseline to gain more insight into the sources of bias.

In sum, this data descriptor addresses the need for a comprehensive, methodologically consistent archive of future changes in multiple measures of evaporative demand from the latest suite of climate projections. We generate CMIP6-derived estimates of ET_P_, ET_0_, and VPD, validate the dataset over the historical period with multiple observational datasets, and conduct a brief evaluation of projected future changes in ET_P_ and ET_0_. This dataset^18^ is built using methods developed for multi-disciplinary hydroclimate and agricultural analyses^13,15^, with the goal of providing the user community a consistent and comprehensive product useful for understanding future changes in water availability. Section 2 describes the methods used to estimate ET_P_, ET_0_, VPD, and other intermediate step variables along with a description of the CMIP6-MME. Section 3 contains a description of where to access and contents of the data repository. Section 4 contains the technical validation of the multiple estimates of evaporative demand and section 5 directs users to the Python codes used to calculate all of the variables described in section 2.

## Methods

The first evaporative demand methodology described and applied herein is the FA0-56 Penman-Monteith formulation (Sec. 2.1) for estimating reference evapotranspiration (ET_0_)^14,16^ which has the most inputs relative to other methods. Within this section we describe the calculation of monthly net surface-radiation (Sec. 2.1.1), 2-meter wind-speed (Sec. 2.1.2), vapor pressure (Sec. 2.1.3), and saturated vapor pressure (Sec. 2.1.4) from both the GCMs and the ERA5-Land reanalysis. Further, we describe the iterations of ET_0_ estimations using two different assumptions of vegetation cover: short grass and alfalfa (Sec. 2.1.5). In Sec. 2.2, we describe the method used to determine the advective and radiation components of ET_0_ and how those components should be interpreted. The subsequent section contains the description of the Priestley-Taylor potential evapotranspiration formulation (ET_P_) (Sec. 2.3) and the terms used in this dataset to generate monthly estimations of ET_P_. The ET_P_ calculated is not split into advective and radiation components due to the choice of coefficient that represents the relative contribution of each component (described further in Sec. 2.3). The final section of the methods (Sec. 2.4) describes the land masking that we apply as a last step for preparation of the data to align with the intended domain of application of the Penman-Monteith and Priestley-Taylor methodologies. Finally, in Sec. 2.5, we describe the set of GCMs were selected based on availability of data and used to generate the CMIP-MME estimates of potential and reference evapotranspiration.

### FAO 56 Penman-Monteith Reference Evapotranspiration

The FAO-56 Penman-Monteith ET_0_ method was developed to estimate the evaporative demand of a well-irrigated crop land, with assumptions about the land-surface vegetation allowing for ET_0_ to be applied in the approximation of required water usage in agricultural operation (R. G. Allen *et al*.^25^; R. G. Allen & Pruitt^16^). It has been applied widely (e.g. Ashofteh Parisa-Sadat *et al*.^26^; Beven^27^; Kay & Davies^28^; Kingston *et al*.^29^; McKenney & Rosenberg^30^; Tabari & Hosseinzadeh Talaee^31^) and is generally considered among the most accurate methods, despite it’s relatively large data requirement^32,33^. Included in the following subsections are descriptions of the ET_0_ method that we applied in the generation of the subject dataset^18^ and all of the intermediate steps required to convert the input variables derived from CMIP6-MME output to represent their form as described in the FA0-56 guidelines. To provide an estimate of the two most commonly applied crop types to the formulation, we generate estimations for both short-grass and alfalfa crops in this dataset^18^ (described further in Sec. 2.1.5).

1 $$E{T}_{0}=\frac{0.408\Delta {R}_{n}+\gamma \left(\frac{{C}_{n}}{T+273}\right)\left({e}_{s}-{e}_{a}\right)}{\Delta +\gamma \left(1+{C}_{d}{u}_{2}\right)}$$

Where ET_0_ is reference evapotranspiration (mm day^−1^), Δ is the slope of the saturated vapor pressure curve (kPa C^−1^), R_n_ is the net radiation (MJ day^−1^), γ is the psychrometric constant (kPa K^−1^), C_n_ and C_d_ are empirically derived values for a given reference crop from Allen *et al*.^25^ (s m^−1^), T is monthly mean air temperature (°C), (e_s_-e_a_) is the saturation vapor pressure deficit (kPa), and u_2_ is the monthly average wind speed at 2 meters (m s^−1^).

### Net Surface-Radiation

Net radiation (R_n_) is calculated here using sensible (hfss) and latent (hfls) heat fluxes from the GCM simulations to implicitly account for the contribution of the ground heat flux (G) to the net radiation balance as is described in Allen *et al*.^25^. This approach is consistent with methods applied in previous evaluations of the surface-energy balance^2^ as follows:

2 $${R}_{n}={\rm{hfss}}+{\rm{hfls}}$$

Though the ground heat flux has been shown in previous studies to be nearly negligible at the monthly time scale^2^, this approach allows us to avoid assumption of zero G in our estimation of ET_0_. We followed this method of using the latent and sensible heat flux here as our analysis using the same category of GCM output data^2^.

### 2-Meter Wind-speed

FAO-56 guidelines call for the use of the surface windspeeds at a height of 2-meter above the land surface^25^, but all CMIP6-MME monthly windspeed output is documented as the 10-meter surface windspeeds. Furthermore, the monthly average 10-meter surface windspeeds we use here are directly computed outputs for each model within the CMIP6 archive (variable name: SfcWind). This approach allows for a more effective capturing of the monthly turbulent flux contribution to ET_0_ as opposed to computing average wind speed from north-south and east-west velocities which yields much smaller magnitude monthly wind speed values. To convert windspeeds to match the specifications of FAO-56, we apply the equation from the FAO-56 guidelines^25^:

3 $${u}_{2}=\frac{4.87{u}_{z}}{{\rm{ln}}\left(67.8z-5.42\right)}$$

Where u_2_ is the 2-meter monthly averaged surface windspeed, u_z_ is the monthly averaged surface windspeed at height z, and z is the height above the land surface expressed in meters of the surface windspeed of the input data, in this case 10 meters.

### Vapor Pressure

Following the guidance of the FAO-56 manual^25^, we estimate the saturated vapor pressure (e_s_) using the monthly mean air temperature from each of the GCMs. Then we utilize e_s_ and the monthly mean relative humidity (h_r_) to calculate a monthly vapor pressure deficit (VPD) and the slope of the saturated vapor pressure curve^25^. Though this variable is an intermediate step in the estimation of the evaporative demand, VPD has utility in a variety of agricultural^34,35^ and ecological^36–38^ applications due to its modulation of stomatal transpiration and relationship with vegetation production, so we have included this variable in the published dataset^18^.

### Saturated Vapor Pressure

The saturated vapor pressure is calculated directly from the monthly mean air temperature (T_avg_) and represents the theoretical pressure of an air mass at saturation for the given T_avg_. It is calculated using formulas from^25,39^:

4 $${e}_{s}=0.6108{e}^{\frac{17.27{T}_{avg}}{{T}_{avg}+237,3}}$$

The FAO56 guidelines suggest that the use of mean of maximum and minimum air temperatures (T_max_ and T_min_ respectively) for use in the saturated vapor pressure on daily timescales is preferable to using daily mean air temperature due to the non-linearity of the diurnal temperature variations, particularly as it can bias the estimation of relative humidity (Allen *et al*.^25^). However, authors tested the use of monthly T_max_ and T_min_ in the estimation of e_s_ and compared with the use of monthly T_avg_ and found marginal differences in the resulting ET_0_ (not shown). This paired with the lack of availability of T_min_ and T_max_ from the CMIP6 archive for all models within the CMIP6-MME used here, it is preferable to use the monthly T_avg_ values in this step.

### Vapor Pressure Deficit (VPD)

VPD is calculated using the relative humidity (h_r_) and e_s_ to estimate the difference between the actual vapor pressure of the air and the saturated vapor pressure of the air on a monthly time scale. VPD is calculated as:

5 $$VPD=\left(1-\frac{{h}_{r}}{100}\right)\ast {e}_{s}$$

### Saturated Vapor Pressure Curve

The final intermediate variable required for both the ET_0_ and ET_P_ (described below) is the slope of the saturated vapor pressure curve (∆). ∆ is the instantaneous rate at which the saturation vapor pressure changes at a given temperature and is calculated as:

6 $$\Delta =\frac{4098{e}_{s}}{{\left({T}_{avg}+237.3\right)}^{2}}$$

### Vegetation Parameters

We apply two formulations of the vegetation parameters that we calibrated for use in the Allen *et al*.^16^ formulation. Guidance from agriculturally focused evaporative demand analyses^40^ suggests that short-grass and alfalfa are the most widely applied crop types for estimation of ET_0_, which is the justification for our selection. Parameter values for C_n_ and C_d_ (Eq. 1) are 900 and 0.34 for respectively for short grass and 1600 and 0.38 for alfalfa^16^. These values represent the vegetation resistance terms within the advective portion of the ET_0_ formulation and were empirically derived in^25^.

### Radiation and Advection components of ET_0_

To evaluate the relative contributions of evaporative demand components to the historical ET_0_ and future changes to ET_0_, we split the numerator of Eq. 1 to serve as close approximations of the radiation (the 0.408ΔRn numerator term) and advection (the $\gamma \left(\frac{{C}_{n}}{T+273}\right)\left({e}_{s}-{e}_{a}\right)$ numerator term) components. One can observe that both terms, ET_0,Rad_ (Eq. 7) and ET_0,Adv_ (Eq. 8) have the same denominator that contains a windspeed term, so it is not to be considered the pure separation of radiation and advection, simply a rough approximation of the relative contribution of each term. When one evaluates the ratio of the two terms, as is done in the technical validation of this manuscript, the advective component that exists in the denominators of the separated components (Eqs. 7, 8) cancel out it then becomes a comparison the relative magnitudes of the radiation and advection terms in the numerator. To be explicit about the equations used, equations of the radiation and advection components of ET_0_ are shown as Eqs. (7, 8) respectively.

7 $$E{T}_{0,Rad}=\frac{0.408\Delta \left(Rn\right)}{\Delta +\gamma \left(1+{C}_{d}{u}_{2}\right)}$$

8 $$E{T}_{0,Adv}=\frac{\gamma \left(\frac{{C}_{n}}{T+273}\right)\left({e}_{s}-{e}_{a}\right)}{\Delta +\gamma \left(1+{C}_{d}{u}_{2}\right)}$$

### Priestley-Taylor

The Priestley-Taylor method is applied for the quantification of monthly radiation-based ET_P_^13^, both for comparison to the ET_0_ method of estimating evaporative demand and to allow for evaluation of projected changes to evaporative demand driven primarily by increased net radiation at the surface. The Priestley-Taylor methodology assumes a constant ratio of the advective and radiative components of ET_P_, therefore its inclusion in this dataset^18^ allows for evaluation of how the increases in surface energy will drive changes to evaporative demand without the inclusion of more uncertain climate parameters such as wind speed or estimates of vegetation contributions. The equation for the Priestley-Taylor formulation of ET_P_ is as follows:

9 $$E{T}_{P}=\frac{\alpha \Delta \left({\rm{Rn}}\right)}{{\rm{Lv}}\left(\Delta +\gamma \right)}$$

Where Δ, Rn, and γ were previously defined in section 2.1 and $\alpha$ is a constant equal to 1.26 empirically derived in^13^. The $\alpha$ coefficient of 1.26, which is most commonly applied to empirically account for the non-radiative components of PET for humid regions^5,13^, is held constant to avoid introducing uncertainty associated with regionalized calibration of the parameter. Other studies have shown that the coefficient can be varied spatially with a higher value generally corresponding to PET estimation in more arid regions^41,42^. If users wish to estimate ET_P_ using distributed coefficients, then we recommend a simple transformation by dividing the entire dataset by 1.26 to which the desired distributed coefficients could be applied.

### Land Masking

The methodologies presented here were explicitly developed for application to land-areas, as opposed to open water areas^25^. Because of this, we mask out all oceans and large water body grid cells using land-sea masks obtained for each GCM, where grid cells containing less than 5% of the total area labelled as land were screened and replaced with null values. We include an archive of the land-sea masks in the published dataset^18^ for the convenience of data users.

### CMIP6-MME

The CMIP6-MME used to generate this dataset^18^ was selected based on the availability of data needed to generate monthly estimations of ET_P_ and ET_0_. Input variables were downloaded from multiple ESGF data nodes at monthly resolution for each of the CMIP6-MME described in Table 1. All data were checked for quality control to screen missing data or data corruption for all input variables. Only one realization from each GCM is used in this dataset^18^, primarily due to the lack of consistency in the number of individual GCM realizations from each modelling organization. The Python code used to generate this dataset^18^ is provided for those who wish to extend this analysis to multiple realizations from individual models.

**Table 1:** Description of each member of the CMIP6-MME including the institution of origin, horizontal resolution of the simulation, and the variant ID used to calculate ET_P_, ET_0_, and VPD.

GCM Name	Institution	Resolution	Variant ID
ACCESS-CM2^48^	Commonwealth Scientific and Industrial Research Organisation	2.8° × 2.8°	r1i1p1f1
ACCESS-ESM1-5^49^			
CESM2^50^	National Center for Atmospheric Research	100 km	
CMCC-CM2^51^	Fondazione Centro Euro-Mediterraneo	1.25° × 0.9°	
CMCC-ESM2^52^	Fondazione Centro Euro-Mediterraneo	100 km	
EC-Earth3^53^	Agencia Estatal de Meteorologia	100 km	
GFDL-ESM4^54^	National Oceanic and Atmospheric Administration, Geophysical Fluid Dynamics Laboratory	100 km	
INM-CM4-8^55^	Institute for Numerical Mathematics	1.4° × 1.4°	
INM-CM5-0^56^	Institute for Numerical Mathematics	1.4° × 1.4°	
IPSL-CM6A-LR^57^	Institut Pierre Simon Laplace	2° × 1.5°	
MIROC6^58^	Atmosphere and Ocean Research Institute, The University of Tokyo and Japan Agency for Marine-Earth Science and Technology	2.5° × 1.3°	
MPI-ESM1-2-HR^59^	Max Planck Institute for Meteorology	250 km	
MPI-ESM1-2-LR^60^	Max Planck Institute for Meteorology Meteorological Research Institute	250 km	
MRI-ESM2-0^61^			
UKESM1-0-LL^62^	Met Office Hadley Centre	1.12° × 1.12°	
HadGEM-GC31-LL^63^	Met Office Hadley Centre	1.9° × 1.25°	r1i1p1f3

Variant IDs are designated as part of CMIP6 guidelines, where each modeling institution labels simulations systematically by realization (r), initialization method (i), physics (p) and forcing (f). The need to use GCM simulations with differing variant IDs arise primarily from data availability. Differences in the forcings (eg. r1i1p1f1 vs r1i1p1f2) of the simulations described below are derived from updated versions of the CMIP6 standardized input forcings but are quantitatively equivalent for each ScenarioMIP experiment in terms of greenhouse gas emissions.

For each of the 16 GCMs in Table 1, monthly estimations of ET_P_, ET_0_, components of ET_0_, and VPD were generated for the historical CMIP6 simulation period (1850–2014) and for the future period of 2015–2099 using four future emission scenarios (SSPs 1–2.6, 2–4.5, 3–7.0. and 5–8.5). The only exception is the lack of a SSP 3–7.0 scenario simulation for the HadGEM3-GC31-LL due to a lack of publication of that specific simulation being published on the ESGF data node. All GCM derived variables contained in the dataset are produced in the native resolution of the parent GCM simulation.

## Data Records

This dataset^18^ contains NetCDF files of estimated ET_P_, multiple estimations of ET_0_ using vegetation parameters for both short-grass and alfalfa, the advective and radiative components of ET_0_, and estimations of VPD that were calculated in the process of estimating ET_0_. All NetCDF files contain information on the date of generation of the dataset, authors of the dataset, variable IDs, input variable information, and attributes of the parent GCM simulations used to generate the estimations of all variables described above. The link to the archived dataset can be found here: 10.5281/zenodo.7789759^18^.

Each NetCDF file contains one variable from one GCM for either the historical simulation or one of the four future emission scenarios that were selected for use in this dataset^18^. The naming convention used for each NetCDF file is consistent with the CMIP6 ESGF naming conventions including variable, frequency, GCM name, scenario, variant ID, grid ID, and date range. Each file for the derived variables within this dataset contains data on the native grid of the parent GCM that was used to generate the estimations described above. Additionally, we include a single land-mask for each individual GCM to clarify which grid cells were selected as land-surface grid cells. Further description of the file structure and organization of data within the dataset^18^ can found in the “dataset_README.txt” file.

## Technical Validation

For the validation of the ET_P_ and ET_0_ estimations across the ensemble of GCMs, we use 30-year means of monthly estimated evaporative demand rates and 30-year mean annual total evaporative demand depth on a grid cell basis for both the GCMs and the historical baseline datasets. The use of long-term means on a grid cell basis permits a validation of the climatic evaporative demand and an evaluation of the spatial coherence of the simulated climate within the ensemble of GCMs when compared to the multiple historical baselines. For this analysis, we independently generate monthly estimates of ET_P_ and ET_0_ from ERA5-Land in order to ensure consistency with our methods, though published hourly estimates of ET_0_^43^ could also serve as a baseline for observationally based historical ET_0_.

The validation period of 1951–1980 coincides with the earliest available data from the ERA5-Land reanalysis and an overlapping period within the historical simulations of the GCMs with minimal acceleration of greenhouse gas emission impacts. We acknowledge that differences in the sensitivities of GCMs within CMIP6^23,24^ could result in the inclusion of GCMs with significant warming even within the period of 1951–1980, but the impact of any warming trends in this early historical period is likely to be minimal because we are considering the long-term mean of ET_P_ and ET_0_ rates.

To compute the spatial correlation and percent bias between estimated fluxes within the GCMs and the observational datasets we align the GCM-simulated evaporative demand fluxes to the 0.5° × 0.5° grid cell resolution of the CRU^21^ and ERA5-Land reanalysis fluxes using a conservative remapping algorithm^44^. The aligning of the GCM derived ET_P_ and ET_0_ is used only for the validation of the dataset^18^ during the historical period and is not a feature of the dataset as published; all GCM estimated evaporative demand terms in the published dataset^18^ remain at the native resolution of the respective GCM simulation.

### Historical Simulations

**Fig. 1:**
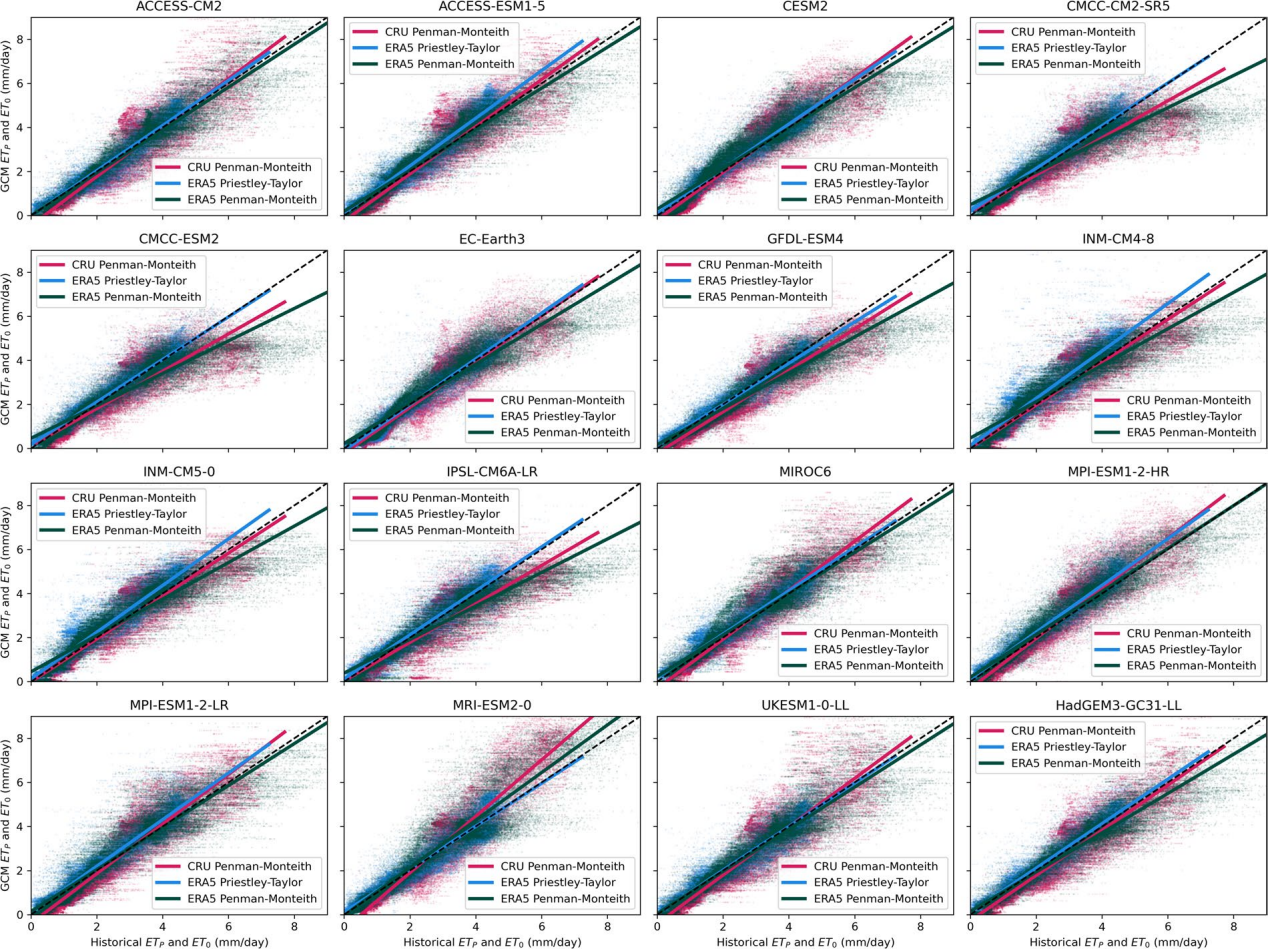
Comparison of GCM ET_P_ and ET_0_ to observational datasets. The CMIP6-MME estimated 30-year mean annual ET_P_ and ET_0_ rates (mm/day) are compared to historical validation datasets (1951–1980) on a grid by grid basis. The resolution of all datasets are spatially adjusted to match the CRU dataset 0.5° × 0.5° resolution for validation, whereas the published datasets retain native GCM resolutions. ET_0_ from GCMs is compared to CRU ET_0_ (magenta) and ERA5-Land ET_0_ (green), whereas ET_P_ estimated from GCMs is compared to ERA5-Land ET_P_ (blue).

Comparisons of the 30-year mean annual rate of ET_P_ and ET_0_ (mm/day) reveal a strong one-to-one relationship between the GCM simulations and historical baselines during the period 1951–1980 (Fig. 1). The global performance of the GCM derived estimations of ET_0_ and ET_P_, is evaluated based on the global average of the 30-year mean annual percent bias (Table 2) and the spatial Pearson correlation^45^ between the 30-year mean annual evaporative demand rates of all land-surface grid cells (Table 3). Validation shown for ET_0_ is based on the short-grass vegetation parameterization within the ET_0_ formulation for brevity, but results for alfalfa vegetation are not qualitatively different from those shown for the short-grass vegetation.

**Table 2:** Percent bias of GCM-based ET_0_ and ET_P_ relative to observational datasets.

	CRU ET_0_	ERA5-Land ET_0_	ERA5-Land ET_P_
ACCESS-CM2	−14.3	−2.4	−7.5
ACCESS-ESM1-5	−9.8	18.2	−1.0
CESM2	−8.5	−0.9	0.2
CMCC-CM2-SR5	−13.4	3.4	−4.5
CMCC-ESM2	−14.7	1.2	−6.0
EC-Earth3	−10.7	−7.0	−2.2
GFDL-ESM4	−19.8	−1.1	−12.7
INM-CM4-8	−6.8	12.2	3.1
INM-CM5-0	−7.4	13.4	2.6
IPSL-CM6A-LR	−13.4	11.8	−5.6
MIROC6	−6.6	5.3	3.5
MPI-ESM1-2-HR	−5.2	18.2	3.5
MPI-ESM1-2-LR	−11.4	8.0	−3.3
MRI-ESM2-0	−5.8	8.1	3.4
UKESM1-0-LL	−13.7	−3.8	−6.1
HadGEM3-GC31-LL	−13.8	0.3	−5.6

The 30-year mean annual percent bias is calculated from the historical baselines for both ET_0_ and ET_P_ over period 1951-1980 of each of the historical CMIP6-MME. For each GCM, the percent bias reported is the mean global percent bias of either ET_0_ or ET_P_ from all land-area grid cells. ET_0_ is compared to both the CRU dataset and the ERA5-Land estimate of ET_0_ generated for this validation.

**Table 3:** Spatial correlation R of ET_0_ and ET_P_ estimated from GCMs compared to observational datasets.

	CRU ET_0_	ERA5-Land ET_0_	ERA5-Land ET_P_
**ACCESS-CM2**	0.92	0.95	0.97
**ACCESS-ESM1-5**	0.93	0.95	0.97
**CESM2**	0.93	0.94	0.97
**CMCC-CM2-SR5**	0.89	0.91	0.96
**CMCC-ESM2**	0.89	0.92	0.97
**EC-Earth3**	0.93	0.95	0.96
**GFDL-ESM4**	0.93	0.95	0.98
**INM-CM4-8**	0.92	0.93	0.94
**INM-CM5-0**	0.93	0.93	0.95
**IPSL-CM6A-LR**	0.9	0.93	0.96
**MIROC6**	0.95	0.94	0.97
**MPI-ESM1-2-HR**	0.94	0.95	0.97
**MPI-ESM1-2-LR**	0.93	0.93	0.96
**MRI-ESM2-0**	0.95	0.94	0.95
**UKESM1-0-LL**	0.92	0.94	0.96
**HadGEM3-GC31-LL**	0.93	0.95	0.97

The spatial Pearson correlation, R, of the 30-year mean annual evaporative demand rate is calculated from the historical baselines for both ET_0_ and ET_P_ over period 1951–1980 of each of the historical GCM simulations. For each GCM, the R reported is the mean global correlation of either ET_0_ or ET_P_ from all land-area grid cells. ET_0_ is compared to both the CRU dataset^21^ and the ERA5-Land estimate of ET_0_ generated for this validation.

The percent bias of the 30-year global mean annual GCM estimates of ET_0_ range from −19.8 to −5.2% when compared to the CRU dataset and from −7.0 to 18.2% compared the ERA5-Land derived estimate (Table 2). ET_P_ from the GCMs demonstrate a range of percent bias from −12.7 to 3.5% for the global mean when compared to the ERA5-Land ET_P_ (Table 2). Visual inspection of the spread of points in Fig. 1. show the largest variations about the one-to-one line are in regions of with the highest historical evaporative demand.

Pearson correlations (R) of the 2-d spatial pattern of 30-year mean annual ET_0_ range from 0.89 to 0.95 when evaluating against the CRU dataset and 0.91 to 0.95 against the ERA5-Land estimated ET_0_ (Table 3). The long-term estimated ET_P_ from the CMIP6-MME and ERA5-Land are even more strongly correlated in the historical period, with R values ranging from 0.94–0.98 (Table 3). The strength of these correlations suggests that, despite the biases in evaporative demand estimations, the GCM simulations all generally reproduce the observed spatial pattern of ET_P_ and ET_0_ across the global land surface.

### FAO-56 Penman-Monteith

**Fig. 2:**
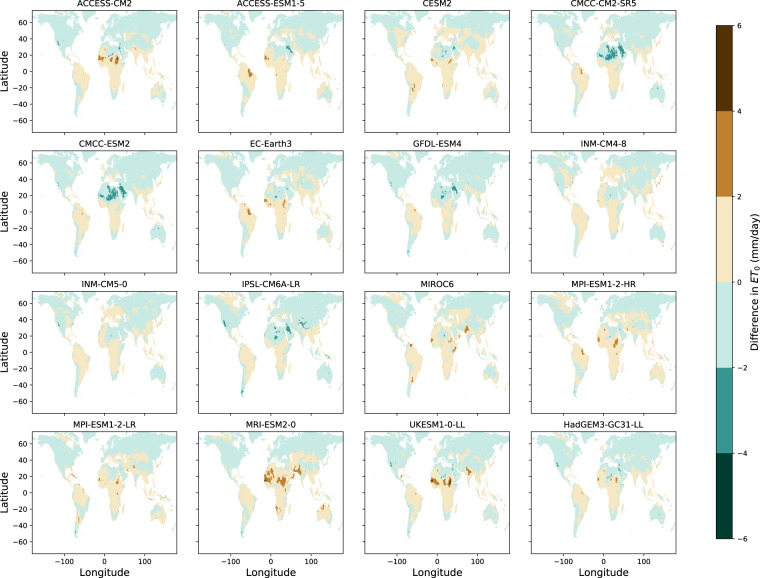
Differences between GCM and CRU ET_0_. CMIP6-MME derived estimations of historical 30-year mean annual short-grass ET_0_ (mm/day) are compared to the CRU historical baseline of ET_0_ over the period (1951-1980) on a grid cell by grid cell basis over the land area. Differences here are in units of (mm/day).

The sign and magnitude of biases of 30-year mean annual ET_0_ reveal some noticeable regional patterns across GCMs. The northern hemisphere appears to have a consistent negative bias for all GCMs (Fig. 2), which is likely the source of the global mean negative ET_0_ bias for the historical period across all 16 GCMs (Table 2) given the majority (~68%) of the land-surface on the Earth is in the northern hemisphere.

Biases relative to the CRU ET_0_ are larger than those relative to the ERA5-Land derived estimates (Table 2) and the R of GCMs estimated ET_0_ is consistently lower when comparing to the CRU ET_0_ versus ERA5-Land derived estimates (Table 3). These differences in the relative agreement of the CMIP6-MME derived estimates are likely due to relatively minor differences in the methods applied to estimate the monthly time series of vapor pressure in CRU’s estimates and the methods used here for both the ERA-Land derived historical baselines and model simulations. Differences in the estimation of vapor pressure deficit between the two datasets arise from differing quantification of relative humidity and different accounting of mean monthly temperatures which are required for estimation of the saturation vapor pressure. When compared the ERA5-Land derived estimates of ET_0_ all GCMs have high spatial correlation (R > 0.91) (Table 3) and only two of the models produce large annual biases: ACCESS-ESM1-5 and MPI-ESM1-2-HR (both 18.2% bias during the historical simulation) (Table 2).

### Priestley-Taylor

Scatter about the one-to-one line exists within all ensembled members of the CMIP6-MME compared to the observational datasets, but the magnitude of the global bias tends to be smaller within the ET_P_ estimations from GCMs compared to the biases of ET_0_. The global multi-model ensemble mean percent bias is −2.3% for ET_P_ from ERA5-Land, the smallest of the ensemble biases compared to the ensemble mean ET_0_ bias of −11.0% for the CRU dataset and 5.3% for the ERA5-Land ET_0_ (Table 2). The smaller range of evaporative demand rates within the ET_P_ estimations compared to ET_0_ (Fig. 1) are consistent with the assumption of a uniform *α* coefficient of 1.26 within the ET_P_ formulation (see Sec. 2.3 description of the coefficient). Others have shown that arid regions should generally have a larger magnitude *α* coefficient applied in the ET_P_ formula^41,42^, which in this case could lead to a more effective representation of the advective component of evaporative demand within those regions. While it is beyond the scope of this dataset^18^ to calibrate spatially distributed *α* coefficients, one could further explore this avenue by performing a transformation of the ET_P_ described in Section 2.3.

**Fig. 3:**
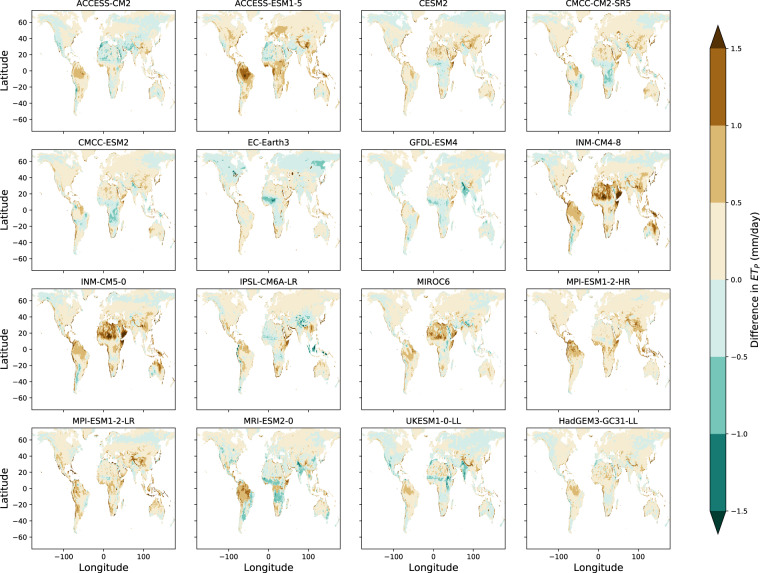
Differences between GCM and ERA5-Land ET_P_. CMIP6-MME derived estimations of historical 30-year mean annual short-grass ET_P_ (mm/day) are compared to the ERA5-Land historical baseline of ET_P_ over the period (1951-1980) on a grid cell by grid cell basis over the land area. Differences here are in units of (mm/day).

Historical ET_P_ from all members of the CMIP6-MME demonstrate positive biases in humid parts of northern South America due simulated net radiation that is much larger in magnitude than in the ERA5-Land reanalysis (Fig. 3). However, this positive bias does not appear to consistently impact regions with high relative humidity climates, as only 8 out 16 GCMs demonstrate the same positive bias within humid regions of Central Africa. Bands of small-magnitude negative ET_P_ bias in the highest northern latitudes are present for 11 out of 16 GCMs, consistent with known differences in the radiative balance of northern-hemisphere ice covered land-areas within the CMIP6-MME compared to observation datasets^46^.

### Advection and Radiation Components of ET_0_

**Fig. 4:**
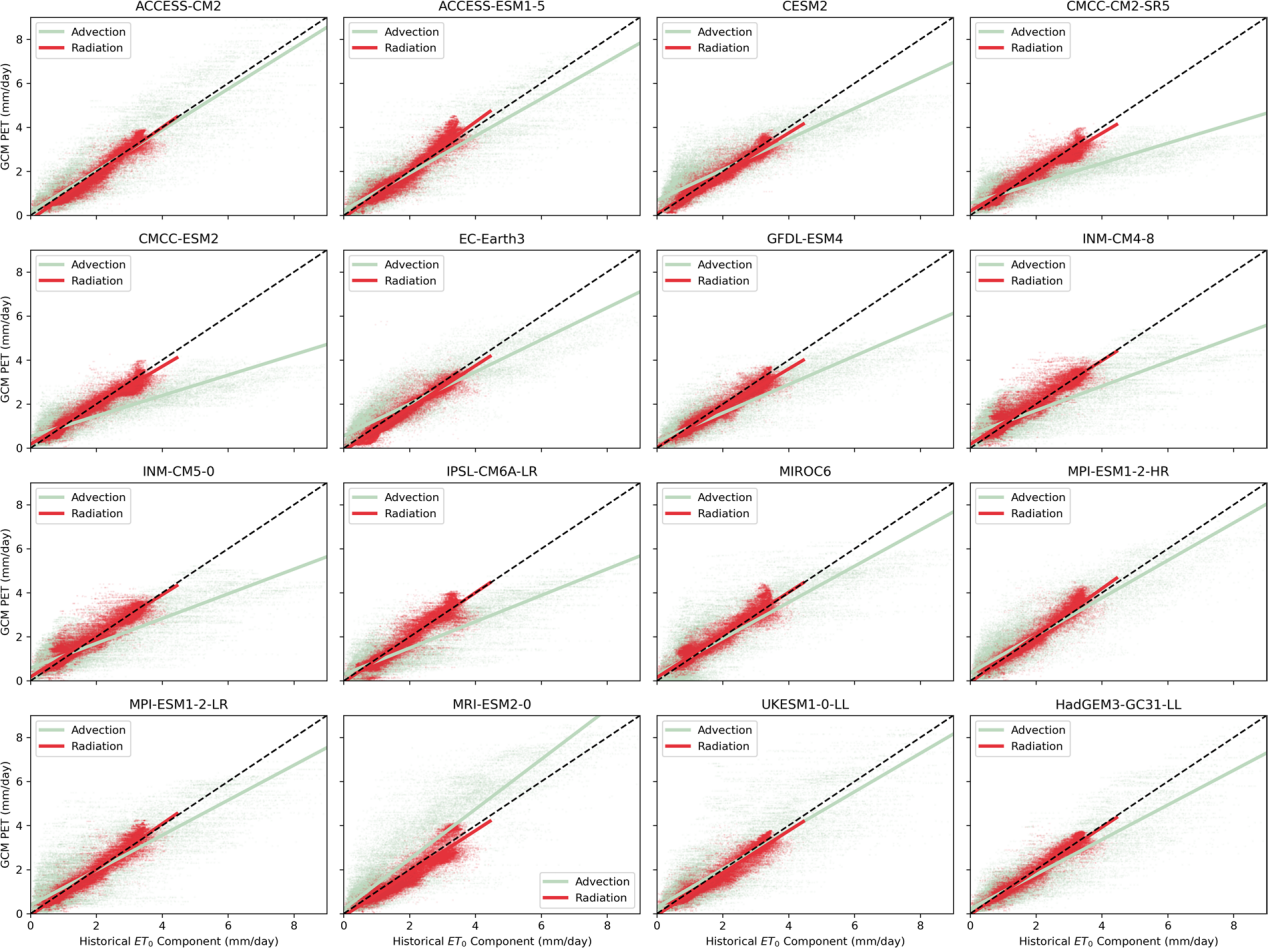
Comparison of advective and radiative components GCM ET_0_ to ERA5-Land. The advective and radiative components of short-grass ET_0_ for each GCM are compared to the estimated components of ET_0_ from ERA5-Land. Each point represents the 30-year mean monthly ET_0_ (mm/day) from 1951–1980 in the historical period for each grid cell across the global land-surface. The advection component (light green) of ET_0_ is primarily modulated by the wind speed and vapor pressure deficit, whereas the radiation component is dependent on the net radiation.

The variations in biases of ET_0_ estimations between GCMs are primarily driven by differences in the biases of the advective component of the evaporative demand. Nine of the 16 GCMs produce both (1) too much advection-driven ET_0 (_ET_0,ADV_) in regions where ERA5-Land has lower magnitude (<3 mm/day) ET_0,ADV_ and (2) too little ET_0,ADV_ in regions of higher magnitude ERA5-Land ET_0,ADV_ (Fig. 4)_._ Only one of the GCMs, MRI-ESM2-0, consistently produces more ET_0,ADV_ than ERA5-Land across all regions (Fig. 4). A potential driver of this bias that exists across multiple GCMs is that the simulated VPD is too large (small) within the regions that demonstrate low (high) levels of advection within ERA5-Land. This hypothesis is supported by examination of the tendency for the nine GCMs that display this bimodal advection bias to underpredict total ET_0_ in more arid regions such as Northern Africa where VPD is known to be high^47^ (Fig. 2). The radiative component of ET_0_ appears to be well-captured by the historical simulations across all 16 GCMs (Fig. 4), which is consistent with the performance of the ET_P_ estimations that are primarily driven by the net radiation (Fig. 1).

## Data Availability

The Python scripts used to calculate ET_P_, ET_0_, ET_0_ components, and VPD can be found within the repository alongside the data described herein. Accompanying the code is a small subset of GCM data that can be used to test run the script. Python scripts utilize a small subset of libraries in the Python3 base and the xarray (v2022.11.0) library to handle calculations of the gridded datasets. The python code used to generate the dataset^18^ described above can be found in the GitHub repository using the following link: https://github.com/nelsbjarke/PET.
